# Real-world effectiveness of doravirine-containing antiretroviral therapy in Chinese adults living with HIV-1: a retrospective study

**DOI:** 10.1186/s12879-026-13533-x

**Published:** 2026-06-10

**Authors:** Hai Long, Xinping Yang, Shenghua He, Jun Liu, Renfang Zhang, Lijun Sun, Hongxin Zhao, Yuanqiu Li, Yaqun Fu, Kathryn Peebles, Yinghui Liu, Fang Sun, Xueyan Liao, Qixin Wang, Fangning Chen, Hao Wu

**Affiliations:** 1 Guiyang Public Health Rescue and Treatment Center, Guiyang, Guizhou Province China; 2 Yunnan Provincial Hospital of Infectious Disease, Kunming, Yunnan Province China; 3 Public Health Clinical Center of Chengdu, Chengdu, Sichuan Province China; 4 Kunming Third People’s Hospital, Kunming, Yunnan Province China; 5 Shanghai Public Health Clinical Center, Shanghai, China; 6 Beijing Youan Hospital, Capital Medical University, Beijing, China; 7 Beijing Ditan Hospital, Capital Medical University, Beijing, China; 8 MSD, Beijing, China; 9 Merck & Co., Inc., Rahway, NJ USA; 10 CO-CRO Medical Development Co., Ltd, Beijing, China; 11 Beijing Key Laboratory for HIV/AIDS Research, Clinical and Research Center for Infectious Diseases, Beijing Youan Hospital, Capital Medical University, Beijing, 100069 China

**Keywords:** Antiretroviral therapy, Doravirine, HIV, Real-world data, Virologic suppression

## Abstract

**Background:**

Doravirine (DOR)-containing antiretroviral therapy (ART) has been recommended as first-line ART by China treatment guidelines since 2021. However, Real-world studies on DOR in China are scarce. We evaluated the real-world effectiveness of DOR-containing ART in Chinese people with HIV-1 (PWH), including treatment-naïve people regardless of viral load (VL) at baseline and treatment-experienced people regardless if they were virologic suppression (VS) or not.

**Methods:**

This was a retrospective, multicenter, observational study using medical chart review in seven hospitals, covering top-tier infectious disease hospitals across regions in China. All the participants who initiated DOR-containing ART during August 2021 and September 2023 were included and followed until September 2024.

**Results:**

At baseline, higher proportions of the 352 participants were male (84.9%), from west region (78.7%), and mean age was 40.0 years. Of the 271 participants with available VL data to confirm the achievement of VS at week 48 ± 8 (271/352), 22.1% were treatment-naïve and 77.9% had prior antiretroviral experience. The adherence rate of DOR in the regimen or DOR/3TC/TDF single tablet was ≥ 80% in 91.5% of participants. Overall, DOR-containing ART achieved a VS rate of 93.7% (95% confidence interval: 90.8%, 96.6%), with rates of 90.0% and 94.8% observed in treatment-naïve and treatment-experienced participants, respectively. Notably, among all six treatment-experienced people who were unsuppressed at baseline, 100% of participants were suppressed at week 48 ± 8. DOR-containing ART appeared to be well tolerated among Chinese PWH, with an adverse event profile consistent with that described in product label.

**Conclusion:**

DOR-containing ART is an effective treatment for Chinese PWH in real-world settings, regardless of their VLs and treatment experience at baseline.

**Clinical trial number:**

EUPAS (registration number: EUPAS103993), registered on [2023-03-23]; NMPA (registration number: CTR20231040), registered on [2023-04-10].

**Supplementary Information:**

The online version contains supplementary material available at 10.1186/s12879-026-13533-x.

## Background

Human immunodeficiency virus type 1 (HIV-1) infection remains a major public health problem. In 2024, there were 1.36 million people with HIV (PWH) in China and the cumulative reported AIDS-related deaths reached 0.49 million [1]. The current HIV treatment in China is specially challenged by the long-term drug toxicity and the consequent treatment discontinuation. Doravirine (DOR), a non-nucleoside reverse transcriptase inhibitor (NNRTI), and its single-tablet combination with lamivudine, tenofovir disoproxil fumarate (DOR/3TC/TDF) have been approved for the treatment of PWH in China in 2020. DOR-containing antiretroviral therapy (ART) has been recommended as first-line ART by China treatment guideline since 2021 [2]. DOR can effectively combat wild type viruses and most common NNRTI resistance mutations [3], with low potential for drug-drug interactions [4]. The non-inferiority of the DOR-containing regimens to ritonavir-boosted darunavir regimens [5] and efavirenz (EFV) based regimens [6], and its non-inferiority in maintaining virologic suppression (VS) after switching from other ART [7] have been confirmed in randomized clinical trials outside China, involving over 100 centers globally but predominantly enrolling White or Black participants. However, clinical trial data of DOR-containing ART in China is scarce and the effectiveness in Chinese PWH, including treatment-naïve and treatment-experienced people in real-world settings is still unclear.

To date, several real-world studies have provided essential external validation for clinical trials and reported the effectiveness of DOR-containing ART among PWH in routine clinical practice. However, four of them had small sample sizes of less than 100 participants [8–11], nine studies included only treatment-experienced or virologically suppressed individuals [12–20], and three studies had follow-up periods of only six months [21–23]. A large-scale retrospective study across 16 sites of five European countries incorporated both ART-naïve and ART-experienced PWH [24]. However, most of the participants were White European, with Asian accounting for only 4.1%. Thus, we conducted a retrospective, multicenter study in China to assess the effectiveness of DOR-containing regimens over 48 ± 8 weeks in real world settings, aiming to fill knowledge gaps regarding DOR using for both treatment-naïve and treatment-experienced people in Chinese population.

## Materials and methods

### Study design and setting

This was a retrospective chart review study in seven centers from five provinces/municipalities (Beijing, Sichuan, Guizhou, Yunnan, and Shanghai), covering top-tier infectious disease hospitals across regions in China. The retrospective data collection started from July 2023 to September 2024 for participants who started DOR-containing regimens from August 2021 (the first participants started DOR-containing ART) until September 2023 (the last participant started DOR-containing ART) at centers, with no interventional procedures involved. The time period was based on expectations of DOR post-marketing period and accrual of eligible participants according to the target sample size. All participants had received ART as part of routine clinical care, and data was generated during routine clinical practice prior to the retrospective chart review process. The study was approved by Institutional Review Boards (IRB) of all centers prior to study execution. For specific IRB information, see the “Ethics approval and consent to participate” section. Participant privacy was well protected, with personal identification data de-identified at the time of analysis and information not provided to entities outside the study.

### Participants

Chinese PWH aged 18 years or older and newly treated with DOR or DOR/3TC/TDF, in accordance with the National Medical Products Administration’s (NMPA) approved product information, were potential subjects for the study. Exclusion criteria included: (1) pregnancy or breast-feeding; (2) participants with documented CrCl < 50 mL/min; (3) participants with documented severe renal/hepatic disease; (4) participants with documented or known resistance to NNRTIs, lamivudine, or tenofovir.

### Procedures and outcomes

Medical chart review was initiated after IRB approval until the overall study enrollment goal was achieved, no matter how many participants for each site had been accrued. Using a standardized case report form, the information was collected by qualified investigators from clinical documentation including but not limited to outpatient or inpatient medical records, laboratory reports, prescription records. Genotypic drug resistance results were extracted from existing reports, primarily using Sanger sequencing with resistance interpretation based on established databases (e.g., Stanford HIVDB). Index date was defined as the date of the first treatment of DOR-containing ART. The primary outcome was VS (viral load [VL, HIV-1 RNA] < 50 copies/mL) at week 48 ± 8 following DOR-containing regimens. Only quantitative measurement or qualitative measurement with a lower limit of detection (LLOD) of 50 copies/mL or lower (e.g., < 40 copies/mL or < 20 copies/mL) was used to determine VS. The effectiveness of DOR-containing ART was defined as the proportion of participants achieving VS at 48 ± 8 weeks among those with available data to confirm the achievement of VS, including participants with confirmed virologic failure before week 40. Virologic failure was defined as persistent plasma VL > 200 copies/mL after 24 weeks of treatment, or ≥ 200 copies/mL after achieving VS and should be designated by investigators and documented in medical records. The adherence of DOR in the regimen or DOR/3TC/TDF single tablet was calculated as the actual number of prescribed pills divided by the total pills that should be prescribed over 48 ± 8 weeks, specifically, for the time interval between the last prescription and HIV viral load testing, the pills prescribed should be regarded as been taken by patients and added in the numerator [25]. An adherence cutoff of at least 80% was used for analyzing the main outcome variable [26]. Treatment discontinuation was defined as a ≥ 60-day gap in DOR-containing ART as documented by the clinician in medical records or calculated as a minimum 60-day gap between the pharmacy refill date and the date when previously dispensed medications were expected to be finished, regardless of whether participants restarted DOR-containing ART after the gap.

### Statistical analysis

Descriptive analyses for continuous variables included mean/median, standard deviation (SD), min/max, interquartile range (IQR, including the first quartile [Q1] and third quartile [Q3]). Frequency and percentage were calculated for categorical variables. Statistical comparisons were conducted using chi-square test for categorical variables, while Student’s t-test, Mann-Whitney U test and Kruskal-Wallis H test were used for continuous variables as appropriate. The 95% confidence intervals (CIs) for binomial proportions were estimated using the Wald’s (normal approximation) method. All available data were used to analyze each variable without imputing missing data. All statistical analyses were performed using SAS 9.4.

Two sensitivity analyses were conducted, including (1) to ensure the closest alignment with the FDA guidance for VS estimation [27], the last available virologic results for the participants with multiple virologic tests at week 48 ± 8 were used for VS evaluation, instead of the results closest to 48-week time point (± 8 weeks); (2) a different adherence cutoff of at least 95% instead of 80% was used for effectiveness evaluation.

### Adverse events/adverse reactions

Adverse events (AEs) and product quality complaints (PQCs) were not actively solicited in this retrospective observational study. The AE or PQC related to DOR and DOR/3TC/TDF identified during chart review were reported. Only AEs with an explicit and definitive notation (by a healthcare provider) of a causal relationship with a product in the medical records or other secondary data should be reported.

## Results

### Baseline characteristics of participants

**Fig. 1:**
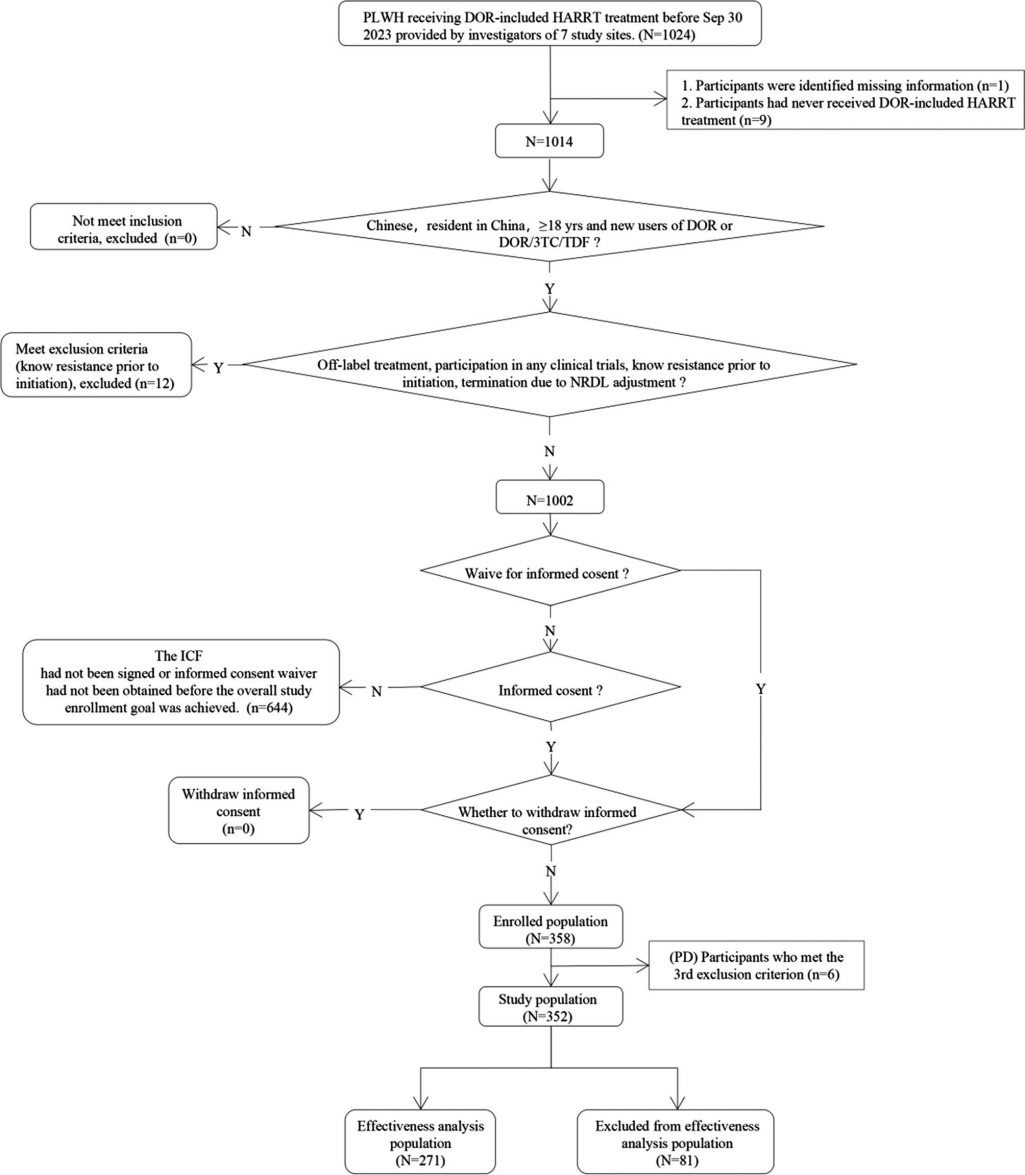
Procedures of eligible participants enrollment. PLWH, participants living with HIV-1; HARRT, highly active antiretroviral therapy; NRDL, national reimbursement drug list; ICF, informed consent form; DOR, doravirine; DOR/3TC/TDF, a single-tablet combination of doravirine, lamivudine and tenofovir disoproxil fumarate. Off-label treatment with DOR-containing ART (exclusion criteria) included: (1) pregnancy or breast-feeding; (2) participants with documented CrCl < 50 mL/min; (3) participants with documented severe renal/hepatic disease

Enrollment procedures of eligible participants were illustrated in Fig. 1. A total of 1024 individuals were admitted to the selected hospitals and started DOR or DOR/3TC/TDF treatment before September 2023. Following the exclusion of individuals who did not provide informed consent and did not meet the criteria for exemption from informed consent, 358 participants were enrolled from August 2021 to June 2024. Next, six participants were further excluded from the study population for pre-existing resistance to NNRTIs and other drugs included in ART after enrolled.

**Fig. 2:**
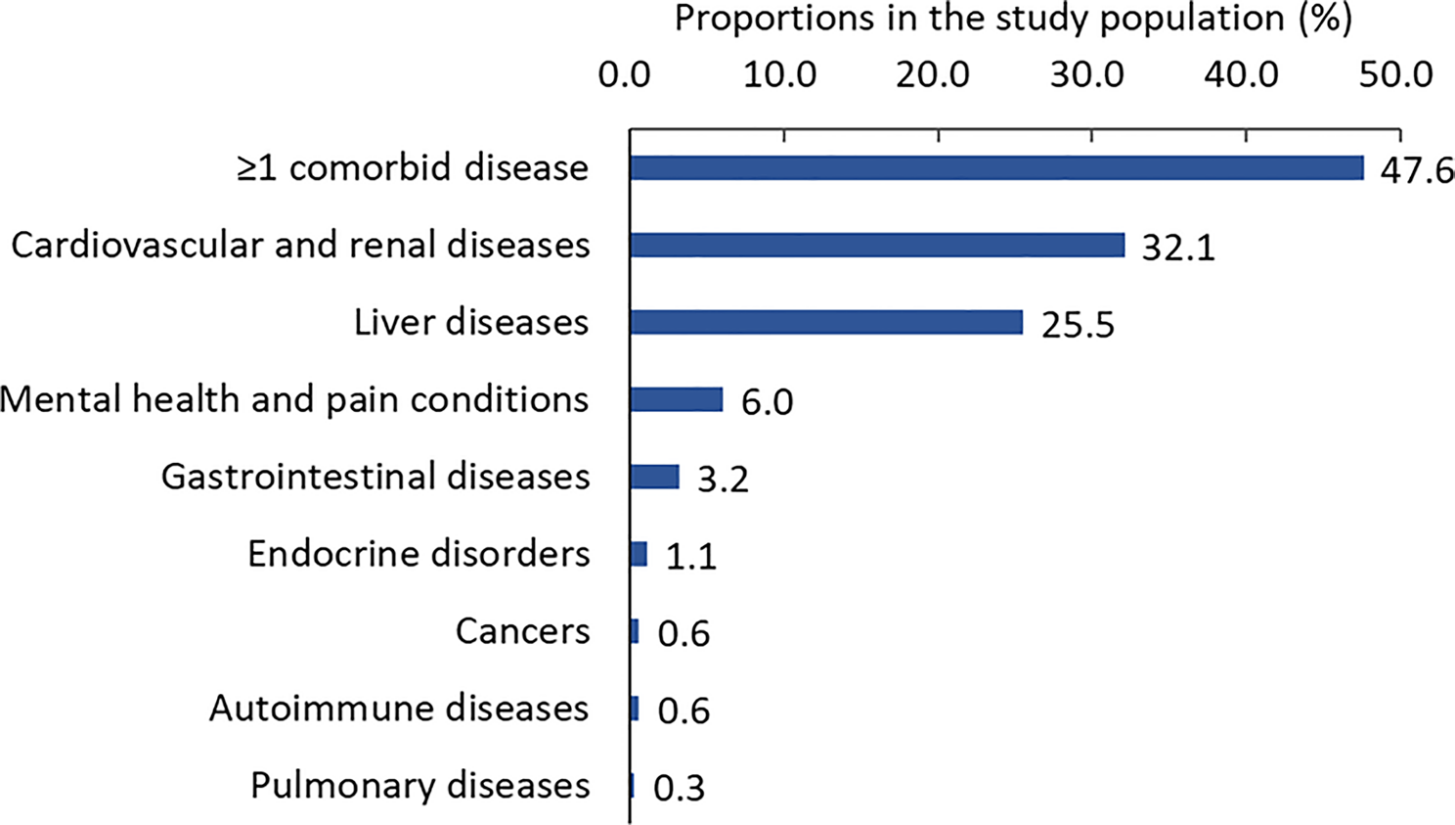
Comorbidities during the observation period in the study population (*N* = 349^*^). ^*^Three participants did not have available comorbidity information during the observation period

The study population comprised 352 participants. The baseline characteristics of the study population are shown in Table 1. Among 352 study population, the mean age at baseline was 40.0 years. Higher proportion of the study population were male (84.9%), from west region (78.7%), covered by public medical insurance (66.8%) and paid by insurance covering DOR/3TC/TDF (87.8%). There were 70 ART-naïve and 282 ART-experienced participants in the study population. Eighty ART-experienced participants were reported to have VL within 30 days prior to the index date, containing a high proportion of VL < 50 copies/mL (92.5%), followed by VL ≥ 50 and ≤ 400 copies/mL (3.8%) and VL > 400 copies/mL (3.8%). Sixty-four ART-naïve participants were reported to have VL within 30 days prior to the index date, containing a high proportion of VL < 100,000 copies/mL (79.7%), followed by VL ≥ 100,000 copies/mL (20.3%). No significant difference in height, weight, smoking, drinking, geographic region and insurance covering DOR/3TC/TDF between the ART-naïve and -experienced participants. Among 349 participants with available comorbidity information during the observation period, 166 (47.6%) had at least one comorbidity. The rates were 40.6% and 49.3% for ART-naïve and -experienced participants, respectively. The most common comorbidities were cardiovascular and renal diseases (32.1%) and liver diseases (25.5%, Fig. 2). No concomitant medications were found during the observation period since only medications that may interact with DOR-containing treatment had been extracted from EMR/paper medical record (not limited to strong cytochrome P450 (CYP) 3 A enzyme inducers).

**Table 1:** Baseline characteristics of the study population (*N* = 352) and effectiveness analysis population (*N* = 271)

Characteristics	Study population (*N* = 352)				Effectiveness analysis population (*N* = 271)			
	Overall (*n* = 352)	TN (*n* = 70)	TE (*n* = 282)	*P* ^a^	Overall (*n* = 271)	TN (*n* = 60)	TE (*n* = 211)	*P* ^a^
Age (years)				0.002				0.002
n (missing)	352 (0)	70 (0)	282 (0)		271 (0)	60 (0)	211 (0)	
Median (Q1-Q3)	37.5 (32.0–48.0)	33.0 (28.0-44.5)	39.0 (33.0-48.8)		37.0 (31.5–47.0)	33.0 (28.0-43.5)	38.0 (32.0–48.0)	
Gender, n (%)				0.565				0.791
n (Missing)	352 (0)	70 (0)	282 (0)		271 (0)	60 (0)	211 (0)	
Male	299 (84.9%)	61 (87.1%)	238 (84.4%)		232 (85.6%)	52 (86.7%)	180 (85.3%)	
Female	53 (15.1%)	9 (12.9%)	44 (15.6%)		39 (14.4%)	8 (13.3%)	31 (14.7%)	
Others	0	0	0		0	0	0	
Height (cm)				0.223				0.229
n (Missing)	291 (61)	68 (2)	223 (59)		235 (36)	58 (2)	177 (34)	
Median (Q1-Q3)	171.0 (167.0-175.0)	170.0 (164.8-174.3)	171.0 (167.0-175.0)		170.0 (165.0-175.0)	170.0 (164.3–174.0)	171.0 (167.0-175.0)	
Weight (kg)				0.685				0.667
n (missing)	328 (24)	68 (2)	260 (22)		255 (16)	59 (1)	196 (15)	
Median (Q1-Q3)	65.0 (59.0–73.0)	65.0 (59.8–70.0)	65.0 (59.0–75.0)		65.0 (59.0–73.0)	65.0 (59.5–70.0)	65.0 (58.8–75.0)	
Smoking, n (%)				0.849				0.934
n (missing)	352 (0)	70 (0)	282 (0)		271 (0)	60 (0)	211 (0)	
Never smoke	77 (21.9%)	14 (20.0%)	63 (22.3%)		54 (19.9%)	10 (16.7%)	44 (20.9%)	
Ex-smoker	7 (2.0%)	1 (1.4%)	6 (2.1%)		5 (1.8%)	1 (1.7%)	4 (1.9%)	
Current smoker	32 (9.1%)	8 (11.4%)	24 (8.5%)		26 (9.6%)	6 (10.0%)	20 (9.5%)	
Unknown	236 (67.0%)	47 (67.2%)	189 (67.1%)		186 (68.7%)	43 (71.6%)	143 (67.7%)	
Alcohol use, n (%)				0.526				0.689
n (missing)	352 (0)	70 (0)	282 (0)		271 (0)	60 (0)	211 (0)	
Never	13 (3.7%)	2(2.9%)	11 (3.9%)		9 (3.3%)	2 (3.3%)	7 (3.3%)	
Ever	7 (2.0%)	0	7 (2.5%)		5 (1.8%)	0	5 (2.4%)	
Currently	43 (12.2%)	11 (15.7%)	32 (11.3%)		33 (12.2%)	9 (15.0%)	24 (11.4%)	
Unknown	289 (82.1%)	57 (81.4%)	232 (82.3%)		224 (82.7%)	49 (81.7%)	175 (82.9%)	
Geographic region^b^, n (%)				0.423				0.337
n (missing)	352 (0)	70 (0)	282 (0)		271 (0)	60 (0)	211 (0)	
East	64 (18.2%)	9 (12.9%)	55 (19.5%)		52 (19.2%)	7 (11.7%)	45 (21.3%)	
Central	8 (2.3%)	1 (1.4%)	7 (2.5%)		5 (1.8%)	1 (1.7%)	4 (1.9%)	
West	277 (78.6%)	59 (84.3%)	218 (77.3%)		213 (78.6%)	52 (86.6%)	161 (76.3%)	
Northeast	3 (0.9%)	1 (1.4%)	2 (0.7%)		1 (0.4%)	0	1 (0.5%)	
Type of medical insurance, n (%)				< 0.001				< 0.001
n (missing)	352 (0)	70 (0)	282 (0)		271 (0)	60 (0)	211 (0)	
Public medical insurance	235 (66.8%)	41 (58.6%)	194 (68.8%)		182 (67.2%)	35 (58.3%)	147 (69.7%)	
Commercial medical insurance	55 (15.6%)	0	55 (19.5%)		39 (14.4%)	0	39 (18.5%)	
Self-pay	58 (16.5%)	29 (41.4%)	29 (10.3%)		47 (17.3%)	25 (41.7%)	22 (10.4%)	
Others	4 (1.1%)	0	4 (1.4%)		3 (1.1%)	0	3 (1.4%)	
Insurance coverage for DOR or DOR/3TC/TDF, n (%)				0.644				0.491
n (missing)	352 (0)	70 (0)	282 (0)		271 (0)	60 (0)	211 (0)	
Yes^c^	309 (87.8%)	60(85.7%)	249 (88.3%)		235 (86.7%)	52 (86.7%)	183 (86.7%)	
No	18 (5.1%)	5 (7.1%)	13 (4.6%)		16 (5.9%)	5 (8.3%)	11 (5.2%)	
Unknown	25 (7.1%)	5 (7.2%)	20 (7.1%)		20 (7.4%)	3 (5.0%)	17 (8.1%)	
Pregnancy, n (%)				1.000				1.000
n (Female)	53	9	44		39	8	31	
Yes^d^	2 (3.8%)	0	2 (4.5%)		1 (2.6%)	0	1 (3.2%)	
No	51 (96.2%)	9 (100.0%)	42 (95.5%)		38 (97.4%)	8 (100.0%)	30 (96.8%)	
Breast-feeding, n (%)				0.416				0.394
n (Female)	53	9	44		39	8	31	
Yes	0	0	0		0	0	0	
No	39 (73.6%)	8 (88.9%)	31 (70.5%)		27 (69.2%)	7 (87.5%)	20 (64.5%)	
Unknown	14 (26.4%)	1 (11.1%)	13 (29.5%)		12 (30.8%)	1 (12.5%)	11 (35.5%)	
VL within 30 days prior to index date of ART-naïve participant, n (%)				-				-
n (missing)	-	64 (6)	-		-	57 (3)	-	
< 100,000 copies/mL	-	51 (79.7%)	-		-	48 (84.2%)	-	
≥ 100,000 copies/mL	-	13 (20.3%)	-		-	9 (15.8%)	-	
VL within 30 days prior to index date of ART-experienced participant, n (%)								-
n (missing)	-	-	80 (202^e^)		-	-	72 (139^f^)	
< 50 copies/mL	-	-	74 (92.5%)		-	-	66 (91.7%)	
≥ 50 and ≤ 400 copies/mL	-	-	3 (3.8%)		-	-	3 (4.2%)	
> 400 copies/mL	-	-	3 (3.7%)		-	-	3 (4.1%)	

^a^*P* values: Comparison between ART-naïve and -experienced participants

^b^The geographic region was categorized as the economic zones defined by the National Bureau of Statistics: https://www.stats.gov.cn/hd/cjwtjd/202302/t20230207_1902279.html

^c^It indicates that DOR or DOR/3TC/TDF have been covered by medical insurance during the participant’s medication process, but it doesn’t mean that the participant has the corresponding medical insurance

^d^It indicates that the participant was pregnant within 12 months prior to index date but wasn’t pregnant on index date

^e, f^Ten and four TE participants had viral load at baseline < 100 copies/mL, respectively. Their VLs were qualitative data without specific values, so they cannot be classified into the below three categories

ART, antiretroviral therapy; TN, ART-naïve participants; TE, ART-experienced participants; Q, quartile; VL, viral load

**Fig. 3:**
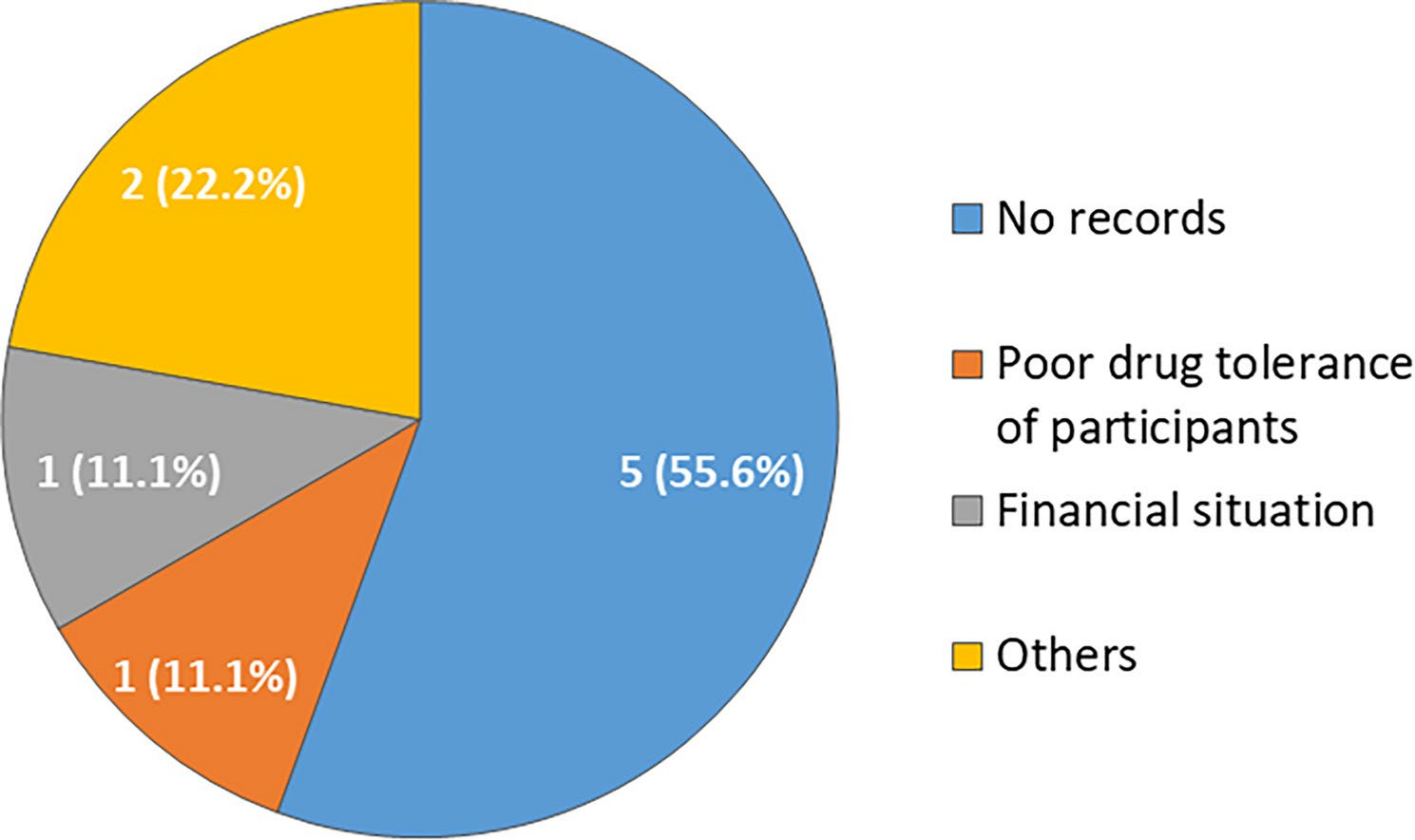
Reasons for discontinuing doravirine-containing treatment in nine participants in the study population

**Fig. 4:**
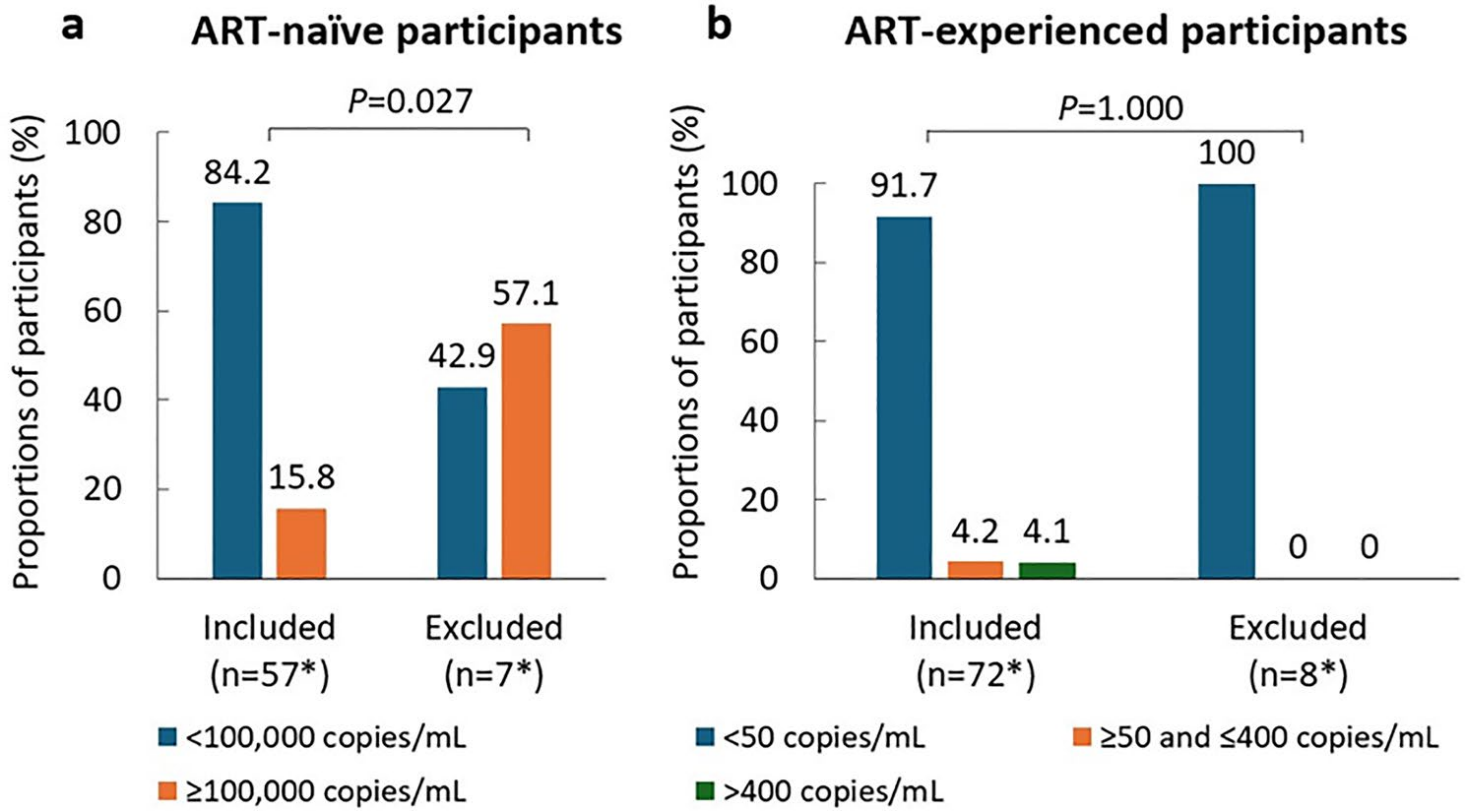
Viral loads within 30 days prior to the index date for the participants included and excluded from the effectiveness analysis (*N* = 271). ^*^For ART-naïve participants, baseline viral loads were missing for three included participants and three excluded participants; For ART-experienced participants, baseline viral loads were missing for 139 included participants and 63 excluded participants. ART, antiretroviral therapy

Among 352 study population, 271 participants (60 ART-naïve and 211 ART-experienced) were included in the effectiveness analysis population since they had available data to confirm the achievement of VS at week 48 ± 8. Among the 81 participants excluded from the effectiveness analysis population, nine discontinued DOR-containing regimens, with reasons shown in Figs. 3 and 68 missed VL data at 48 ± 8 weeks, and four had unqualified HIV-1 RNA tests at week 48 ± 8 with the LLOD > 50 copies/mL. Baseline characteristics of the effectiveness analysis population were shown in Table 1. There were no significant differences in demographic and clinical characteristics between participants included and excluded from the effectiveness analysis population. However, The proportion of VL < 100,000 copies/mL within 30 days prior to the index date in the ART-naïve participants included in the effectiveness analysis was higher than that in the ART-naïve participants excluded in the effectiveness analysis (84.2% vs. 42.9%, *P* = 0.027, Fig. 4). Among the six ART-experienced participants with unsuppressed VLs at baseline in effectiveness analysis population, three had drug resistance testing and showed no drug resistance, the other three did not receive drug resistance testing due to the baseline VLs under 200 copies/mL and other unknown reasons (e.g., participants willingness).

### Antiretroviral therapy regimens and drug resistance

Regarding the regimens prior to index date in the effectiveness analysis population, 44 of 211 (20.9%) ART-experienced participants had at least one change in combination ART (cART) regimens during 1-year prior to index date. The most common cART regimens before switching to DOR-containing regimens were dual nucleoside reverse transcriptase inhibitor (NRTI) + NNRTI (81.0%), followed by dual NRTI/ NRTI + integrase strand transfer inhibitor (INSTI, 10.9%, Table 2). Among the 196 (92.9%) participants with the recorded date of treatment initiation, the duration of the last cART regimen before index date ranged from less than one year to 13.4 years (median 1.3 years). For the DOR-containing regimens, most participants (98.9%) were administrated with DOR/3TC/TDF, and only three participants (1.1%) were treated with DOR and DOR/3TC/TDF in different periods. No participants only used DOR for 48 ± 8 weeks. The proportion of participants with adherence ≥ 80% was 91.5% (248/271).

**Table 2:** The combination antiretroviral therapy regimen before switching to doravirine-containing treatment

cART regimens	TE in effectiveness analysis population (*n* = 211), n (%)	TE in study population (*n* = 282), n (%)
Dual NRTI+NNRTI^a^	171 (81.0%)	220 (78.0%)
Dual NRTI + PI	14 (6.6%)	22 (7.8%)
Dual NRTI/NRTI+INSTI	23 (10.9%)	36 (12.8%)
PI+INSTI	0	0
Others	3 (1.5%)	4 (1.4%)

^a^NNRTIs (non-nucleoside Reverse Transcriptase Inhibitor) before switching to DOR-containing treatment including efavirenz (EFV), ainuovirine (ANV), and nevirapine (NVP)

cART, combination antiretroviral therapy; TE, ART-experienced; NRTI, nucleoside reverse transcriptase inhibitor; INSTI, integrase strand transfer inhibitor; PI, protease inhibitor

A total of 42 (11.9%) participants had at least one genotypic drug resistance testing prior to index date in the study population and showed no resistance to NNRTIs and other ART drugs (Table 3). The NNRTI mutations identified in two of the individuals excluded from the study population were V179E and E138K, with mutations of other excluded participants not reported. Meanwhile, 18 (5.1%) participants had at least one genotypic drug resistance test during the observation period in the study population, with four participants showing genotype-specific drug resistance. Of the four participants, two ART-naïve participants underwent drug resistance testing after 24 weeks of DOR-containing ART and showed resistance to EFV and nevirapine (NVP) (this was documented in the resistance testing report, but the exact mutations were not described). However, one showed no genotype mutations associated with DOR resistance, and the other one was not tested for DOR resistance. As they had not previously received EFV and NVP, the observed resistance was probably due to pre-existing primary NNRTI resistance-associated mutations. The two NNRTI-resistant participants did not achieve VS at week 48 ± 8 with VLs of 50–200 copies/mL. The other two ART-experienced participants were resistant to protease inhibitors (PIs, including nelfinavir [NFV] and tipranavir/ritonavir [TPV/r]) and NRTIs (stavudine [D4T], didanosine [DDI], abacavir [ABC], emtricitabine [FTC], 3TC, TDF), respectively, with exact mutations not described in the resistance test report. However, they received genotypic drug resistance testing within one week after the index date and should be considered as having drug resistance at baseline. The participants with resistance to PIs achieved VS at week 48 ± 8 and the participants with resistance to NRTIs have no available virologic result at week 48 ± 8. None of the four participants discontinued DOR or DOR/3TC/TDF treatment.

**Table 3:** ART drug resistance prior to index date and during the observation period in the study population (*N* = 352)

	ART-naïve participants (*n* = 70)	ART-experienced participants (*n* = 282)	Study population (*n* = 352)
Genotypic drug resistance testing prior to index date			
Total number^a^	70	282	352
Having at least one testing, n (%)	13 (18.6%)	29 (10.3%)	42 (11.9%)
Total number^a^	13	29	42
Having at least one drug resistance, n (%)	0	0	0
Genotypic drug resistance testing during the observation period			
Total number^a^	70	282	352
Having at least one testing, n (%)	12 (17.1%)	6 (2.1%)	18 (5.1%)
Total number^a^	12	6	18
Having at least one drug resistance, n (%)	2 (16.7%)	2^b^ (33.3%)	4 (22.2%)
Resistance to NNRTIs, n (%)	2 (16.7%)	0	2 (11.1%)
Resistance to NRTIs, n (%)	0	1 (16.7%)	1 (5.6%)
Resistance to PIs, n (%)	0	1 (16.7%)	1 (5.6%)

^a^The total number is the denominator for the proportions presented below

^b^The two participants received genotypic drug resistance testing within one week after the index date and should be considered as having tests at baseline

ART, antiretroviral therapy; NRTI, nucleoside reverse transcriptase inhibitor; NNRTI, non-nucleoside reverse transcriptase inhibitor; INSTI, integrase strand transfer inhibitor; PI, protease inhibitor; VS, virologic suppression

Notably, among the other 14 participants without drug resistance, 10 received drug resistance testing within one week after the index date. Of these 10 participants, eight achieved VS at week 48 ± 8, one had no virologic data available at week 48 ± 8, and one had two VL testing results at week 48 ± 8 (one was 64.3 copies/mL, and another one was target not detected [TND]), which might be considered as blips. Four participants received drug resistance testing after 24 weeks. Of them, two had no available virologic data at week 48 ± 8, one could not be regarded as VS or not because the assay had a LLOD of 250 copies/mL at week 48 ± 8, and one achieved VS (< 20 copies/mL) at week 48 ± 8. This indicates that participants who had evidence of no drug resistance no matter at baseline or during the treatment process were likely to achieve satisfactory therapeutic effects after 48 weeks of treatment.

### Effectiveness of DOR-containing regimens

In the effectiveness analysis population, 254 of 271 participants (93.7%, 95% CI: 90.8%, 96.6%) achieved VS at week 48 ± 8. Among the 17 participants not achieving VS, 14 (82.4%) participants exhibited VLs of 50–200 copies/mL, one (5.8%) participant had a VL of 285 copies/mL, and two (11.8%) participants had VL > 1000 copies/mL (19,959 and 26,500 copies/mL) at week 48 ± 8 with low adherence rates of 54% and 64%, respectively, and no available baseline VL data (Table 4). No virologic failure before week 40 was documented in electronic/paper medical records. When analyzing only the 248 participants with adherence ≥ 80% during the observation period, the VS rate at week 48 ± 8 was 94.0% (233/248, 95% CI: 91.0%, 96.9%), with 15 participants not achieving VS as previously described (Table 4).

**Table 4:** Effectiveness of DOR-containing ART at week 48 ± 8

	Participants, *n* (%^a^)	95% CI^b^
VL for effectiveness analysis population (*n* = 271)		
< 50 copies/mL	254 (93.7%)	(90.8%, 96.6%)
≥ 50copies/mL	17 (6.3%)	(3.4%, 9.2%)
≥ 50copies/mL and ≤ 200copies/mL	14 (5.2%)	(2.5%, 7.8%)
> 200copies/mL	3 (1.1%)	(0.0%, 2.4%)
VL for adherence ≥ 80% (*n* = 248)		
< 50 copies/mL	233 (94.0%)	(91.0%, 96.9%)
≥ 50copies/mL	15 (6.0%)	(3.1%, 9.0%)
VL for ART-naïve participants (*n* = 60)		
< 50 copies/mL	54 (90.0%)	(82.4%, 97.6%)
≥ 50copies/mL	6 (10.0%)	(2.4%, 17.6%)
VL for ART-experienced participants (*n* = 211)		
< 50 copies/mL	200 (94.8%)	(91.8%, 97.8%)
≥ 50copies/mL	11 (5.2%)	(2.2%, 8.2%)
VL for ART-naïve and ART-experienced participants without VS before DOR initiation (*n* = 66)		
< 50 copies/mL	60 (90.9%)	(84.0%, 97.8%)
≥ 50 copies/mL	6 (9.1%)	(2.2%, 16.0%)
VL for ART-experienced participants with VS before DOR initiation (*n* = 66)		
< 50 copies/mL	62 (93.9%)	(88.2%, 99.7%)
≥ 50 copies/mL	4 (6.1%)	(0.3%, 11.8%)

^a^The denominator of proportions is the number of participants in the specific category

^b^The 95% CI for a binomial proportion were estimated using the Wald’s (Normal Approximation) method

CI, confidence interval; VL, viral load; VS, virologic suppression; ART, antiretroviral therapy; DOR, doravirine

When classifying participants by ART experience regardless if they were VS or not at baseline, 90.0% (54/60, 95% CI: 82.4%, 97.6%) of ART-naïve participants and 94.8% (200/211, 95% CI: 91.8%, 97.8%) of ART-experienced participants achieved VS at week 48 ± 8 (Table 4). Among the six naïve participants not achieving VS (all had adherence ≥ 80%), none had genotypic drug resistance testing at baseline, while two underwent drug resistance testing at week 24 of DOR-containing ART and were found resistant to EFV and NVP (the exact mutations were not described in the resistance testing report) but not DOR (one was sensitive to DOR and the other was not tested for DOR resistance). Among the 11 ART-experienced participants not achieving VS at 48 ± 8 weeks, only two participants had drug resistance testing at baseline and showed no resistance to NNRTIs. Among these two participants, one lacked baseline VL data with an adherence of 64%, while the other had baseline VL < 50 copies/mL and 100% adherence. Therefore, only one of 11 ART-experienced participants not achieving VS ultimately had clear evidence of baseline VL < 50 copies/mL and no NNRTI resistance.

Among the 66 participants who were ART-naïve or ART-experienced without VS before DOR initiation, 60 (90.9%, 95% CI: 84.0%, 97.8%) participants achieved VS at week 48 ± 8 (Table 4). Of the 66 ART-experienced participants with confirmed VS before DOR initiation, 62 (93.9%, 95% CI: 88.2%, 99.7%) participants achieved VS at week 48 ± 8 (Table 4).

### Sensitivity analysis for the effectiveness of DOR-containing regimens

The study conducted two sensitivity analyses: (1) Using the last available virologic result rather than the one closest to the 48-week point resulted in one more participant achieving VS and an overall effective rate of 94.1% (255/271, 95% CI: 91.3%, 96.9%); (2) Among the participants with adherence ≥ 95%, 94.0% (204/217, 95% CI: 90.9%, 97.2%) achieved VS at week 48 ± 8. Only one participant with adherence ≥ 95% had a VL > 200 copies/mL at week 48 ± 8 (285 copies/mL).

### Adverse events and adverse reactions

Overall, DOR-containing ART was well tolerated. During the study period, 26 AEs from 18 participants (5.1%) were reported. Abnormal hepatic function (6/26) or hepatic failure (2/26) were reported as the most frequent AEs, followed by insomnia (3/26), dizziness (2/26), diarrhoea (2/26), hyperuricaemia (2/26), neutrophil count decreased (2/26), and others reported once (blood glucose increased, nausea, creatinine renal clearance decreased, protein urine present, abnormal dreams, haematuria, hyperlipidaemia). There were no PQCs (with or without AE), special situations (regardless of causality), or spontaneously reported AEs/PQCs for DOR or DOR/3TC/TDF observed in this study.

All of the AEs reported among participants were non-serious events, only 2 were moderate, the rest (*n* = 24) were mild. Among these 26 AEs, 17 events were recovering/ recovered, and 9 events were not recovered. No relevant clinical symptoms were reported for all the cases of abnormal hepatic function or hepatic failure.

## Discussion

In this multicenter, real-world, retrospective study of 271 Chinese adults living with HIV-1, with most of ART-naïve and ART-experienced individuals having baseline VLs < 100,000 copies/ml, DOR-containing ART achieved a VS rate of 93.7% after 48 ± 8 weeks of treatment, with 90.0% and 94.8% effectiveness in ART-naïve and ART-experienced participants, respectively. No virologic failure was documented in electronic/paper medical records during the observation period. DOR-containing regimens were well tolerated, with no serious AEs or safety signals identified in clinical practice.

The effectiveness of DOR-containing regimens in this Chinese real-world study was similar to or higher than that in pivotal worldwide clinical trials, no matter for treatment-naïve participants (84% for DOR in DRIVE-FORWARD [5], 84.3% for DOR/3TC/TDF in DRIVE-AHEAD [6]), or for treatment-experienced and virologically suppressed participants (90.8% for DOR/3TC/TDF in DRIVE-SHIFT [7]). The different results between this real-world study and clinical trials may be partially explained by different adherence measures and administration, data availability of baseline genotypic drug resistance, and the definition of treatment failure, etc. These factors are inherent limitations of real-world studies, making direct comparisons with clinical trials difficult. Nevertheless, our study further showed the effectiveness of DOR-containing regimens in real-world clinical practice, beyond well-designed clinical trials with carefully selected subjects.

The results were consistent with other real-world studies. High rates of VL < 50 copies/mL at week 48 following DOR-containing regimens were observed in both ART-naïve (87.3%) and ART-experienced (91.2%) PWH in routine clinical practice across five European countries [24]. In a UK retrospective study based on chart review, 90% (9/10) of ART-naïve participants and 95% (244/256) of ART-experienced participants treated with DOR-containing ART had VL < 50 copies/mL at six months [22]. Among 50 ART-experienced French participants with long-term VS, 98.0% maintained VS and restored CD4 + T cell count at week 48 after switching from three-drug regimens to DOR/3TC regimens [11]. A retrospective study of 52 Italian participants observed reduced lipid levels and 94.3% VS at week 24 after switching to DOR-containing ART [13]. A national prospective study in the Netherlands reported a long-term non-inferiority of switching to DOR-based ART for two years relative to continuing non-DOR regimens among well-suppressed participants in a real-world setting [12]. The recently published single-center real-world study for 167 PWH in China reported VS rates of 91.3% among ART-naïve participants and 97.6% among ART-experienced participants at week 24 of DOR/3TC/TDF treatment. Notably, in our study, DOR-containing ART was highly effective in six treatment-experienced but virologically unsuppressed participants, all of whom achieved VS at week 48 ± 8.

Although AEs and PQCs were not actively solicited in this study, there are certain circumstances in which individual AEs and/or PQCs must be reported. For example, during review of medical records or physician notes (paper or electronic), to collect data as required by the protocol, if a notation is identified, the AE/PQC must be reported. As shown in this secondary data collection process, similar abnormalities of hepatic (e.g., increased ALT, AST, ALP, total bilirubin, etc.) indicators were also reported in two previous phase III trials (DRIVE-FORWARD & DRIVE-AHEAD) according to the local label. Other frequently reported AEs such as insomnia, dizziness, and diarrhea, were also already reported in previous pivotal studies and described in local label. No potential safety signal was identified in this study.

We provided the most comprehensive and robust characterization of the effectiveness and tolerability of DOR-containing regimens in China, based on the large sample size from different clinical settings. Unlike tightly controlled clinical trials, this non-interventional real-world study used medical chart reviews and better reflects how DOR-containing regimens are prescribed and perform in routine clinical practice. Compared to clinical trials, the inclusion and exclusion criteria for this study were broader and consistent with the population specified in the instructions of DOR-containing prescription in China. Moreover, we addressed knowledge gaps in previous real-world studies by including more treatment-naïve participants and not limiting the prior VL of treatment-experienced participants.

This study had several limitations. With this retrospective study design, VS rates might be over- or underestimated due to factors such as adherence, persistence, possible unknown drug resistance. A higher proportion of treatment-naïve participants had baseline VL < 100,000 copies/mL compared to those excluded from effectiveness analysis due to missing 48-week VL data. This imbalance may have introduced selection bias, potentially favoring higher observed VS rates. Baseline VS status could not be confirmed for 52.4% (142/271) of the effectiveness analysis population, especially for ART-experienced participants, which may complicate the interpretation of overall VS rate. Nonetheless, the subgroup analysis (ART-naïve and -experienced participants without VS before DOR initiation vs. ART-experienced participants with VS before DOR initiation) revealed similar VS rates exceeding 90%. Moreover, genotypic resistance testing data were limited at baseline (11.9%) and during observation period (5.1%), potentially including participants with NNRTI or other ART resistance, introducing uncertainty into the observed effectiveness estimates, and complicating interpretation of unsuccessful treatment outcomes. Another limitation was that we didn’t collect the reason for switching to DOR which is helpful for better understanding the treatment environment and informing treatment strategies. The inherent limitation of retrospective study design may restrict investigators from reminding participants to take their medicines timely. Prescription monitoring may not reflect actual medication adherence. One of the main strengths of this multicenter study was the large sample size of participants in real-world clinical settings rather than clinical trials, with unselective identification and enrollment of all eligible participants who consented or were exempted by IRB to minimize selection bias. Altogether, this strictly supervised study with large sample size provided valuable nationwide real-world evidence of the efficacy of DOR-containing regimens in China.

In conclusion, this is the largest post-marketing observational study conducted in China to assess the effectiveness of DOR included in ART in Chinese PWH. DOR-containing ART has shown high overall effectiveness in ART-naïve participants regardless of VL at baseline and ART-experienced participants regardless if they were VS or not, consistent with the findings of previous overseas real-world studies. Continued monitoring of the prescription patterns and effectiveness of DOR-containing ART in different regions of China is needed to better inform clinical decision-making and optimize its application in practice.

## Supplementary Information

Below is the link to the electronic supplementary material.

Supplementary Material 1: Supplementary Table 1. Ethics Approval. This table lists the name of the IRBs and the approval numbers for Protocol V1.0 and V2.0.

## Data Availability

The data of participants living with HIV included in this study are not publicly available due to their sensitive and confidential nature. The study protocol can be available from whdoc@sina.com upon reasonable request after publication. Researchers interested in collaboration may contact the corresponding author for further details.

## Abbreviations

DOR: Doravirine
ART: Antiretroviral therapy
PWH: People with HIV
VL: Viral load
VS: Virologic suppression
HIV-1: Human immunodeficiency virus type 1
NNRTI: Non-nucleoside reverse transcriptase inhibitor
DOR/3TC/TDF: Single-tablet combination of doravirine, lamivudine, and tenofovir disoproxil fumarate
EFV: Efavirenz
IRB: Institutional Review Boards
NMPA: National Medical Products Administration
LLOD: Lower limit of detection
SD: Standard deviation
IQR: Interquartile range
CI: Confidence interval
AE: Adverse events
PQC: Product quality complaint
CYP: Cytochrome P450
cART: Combination antiretroviral therapy
NRTI: Nucleoside reverse transcriptase inhibitor
INSTI: Integrase strand transfer inhibitor
NVP: Nevirapine
PI: Protease inhibitor
NFV: Nelfinavir
TPV/r: Tipranavir/ritonavir
D4T: Stavudine
DDI: Didanosine
ABC: Abacavir
FTC: Emtricitabine
TND: Target not detected
TN: ART-naïve participants
TE: ART-experienced participants
