# Supplementary material for: Real-world effectiveness of doravirine-containing antiretroviral therapy in Chinese adults living with HIV-1: a retrospective study
Source: BMC Infect Dis. 2026 Jun 10;26:1223. doi: 10.1186/s12879-026-13533-x (PMC13330233; doi:10.1186/s12879-026-13533-x)
Supplement: Supplementary file 1 — Supplementary Material 1: Supplementary Table 1. Ethics Approval. This table lists the name of the IRBs and the approval numbers for Protocol V1.0 and V2.0. [file 12879_2026_13533_MOESM1_ESM.docx]

# Supplementary Tables

Supplementary Table 1 Ethics Approval

| Name of Ethics Committee | Principal Investigator | Approval No. for Protocol V1.0 | Approval No. for Protocol V2.0 |
| --- | --- | --- | --- |
| Beijing Youan Hospital, Capital Medical University | Hao Wu | JYKLZ[2022]132 | JYKLZ[2023]053 |
| Beijing Ditan Hospital, Capital Medical University | Hongxin Zhao | Not available (the Protocol had been updated to V2.0 at the time of site initiation). | DTEC-YW2023-023-01 |
| Shanghai Public Health Clinical Center | Renfang Zhang | GWLS2023-E005-01 | 2023-E005-02 |
| Public Health Clinical Center of Chengdu | Shenghua He | YJ-K2023-01-01 | YJ-K2023-01-02 |
| Yunnan Provincial Hospital of Infectious Disease | Xinping Yang | Not available (the Protocol had been updated to V2.0 at the time of site initiation). | 2023-LS-12 |
| Kunming Third People’s Hospital | Jun Liu | KSLL20230320002 | KSLL20230320002-01 |
| Guiyang Public Health Rescue and Treatment Center | Hai Long | (2023)LSD(01) | (2023)LSD(01--02) |
